# Insecticidal Activity of Organic Extracts of *Solidago graminifolia* and Its Main Metabolites (Quercetin and Chlorogenic Acid) against *Spodoptera frugiperda*: An In Vitro and In Silico Approach

**DOI:** 10.3390/molecules27103325

**Published:** 2022-05-22

**Authors:** Verónica Herrera-Mayorga, José Alfredo Guerrero-Sánchez, Domingo Méndez-Álvarez, Francisco A. Paredes-Sánchez, Luis Víctor Rodríguez-Duran, Nohemí Niño-García, Alma D. Paz-González, Gildardo Rivera

**Affiliations:** 1 Laboratorio de Botánica, Unidad Académica Multidisciplinaria Mante, Universidad Autónoma de Tamaulipas, El Mante 89840, Mexico; evherrera@docentes.uat.edu.mx (V.H.-M.); a2173520279@alumnos.uat.edu.mx (J.A.G.-S.); faparedes@docentes.uat.edu.mx (F.A.P.-S.); luis.duran@docentes.uat.edu.mx (L.V.R.-D.); nngarcia@docentes.uat.edu.mx (N.N.-G.); 2 Laboratorio de Biotecnología Farmacéutica, Centro de Biotecnología Genómica, Instituto Politécnico Nacional, Reynosa 88710, Mexico; doomadv@hotmail.com (D.M.-Á.); delpaz11@gmail.com (A.D.P.-G.)

**Keywords:** chlorogenic acid, insecticide, quercetin, *Spodoptera frugiperda*, *Solidago graminifolia*

## Abstract

*Spodoptera frugiperda* (*S. frugiperda*) remains a global primary pest of maize. Therefore, new options to combat this pest are necessary. In this study, the insecticidal activity of three crude foliar extracts (ethanol, dichloromethane, and hexane) and their main secondary metabolites (quercetin and chlorogenic acid) of the species *Solidago graminifolia* (*S. graminifolia*) by ingestion bioassays against *S. frugiperda* larvae was analyzed. Additionally, the extracts were phytochemically elucidated by ultra-performance liquid chromatography-mass spectrometry (UPLC-MS) analysis. Finally, an in silico study of the potential interaction of quercetin on *S. frugiperda* acetylcholinesterase was performed. Organic extracts were obtained in the range from 5 to 33%. The ethanolic extract caused higher mortality (81%) with a half-maximal lethal concentration (LC_50_) of 0.496 mg/mL. Flavonoid secondary metabolites such as hyperoside, quercetin, isoquercetin, kaempferol, and avicularin and some phenolic acids such as chlorogenic acid, solidagoic acid, gallic acid, hexoside, and rosmarinic acid were identified. In particular, quercetin had an LC_50_ of 0.157 mg/mL, and chlorogenic acid did not have insecticidal activity but showed an antagonistic effect on quercetin. The molecular docking analysis of quercetin on the active site of *S. frugiperda* acetylcholinesterase showed a −5.4 kcal/mol binding energy value, lower than acetylcholine and chlorpyrifos (−4.45 and −4.46 kcal/mol, respectively). Additionally, the interactions profile showed that quercetin had π–π interactions with amino acids W198, Y235, and H553 on the active site.

## 1. Introduction

Plants regulate the production, release, and biological action of secondary metabolites as a defense strategy in natural interactions (i.e., herbivory). It is known that these natural products have been used to treat up to 87% of human diseases; however, there is a lack of phytochemical, pharmacological and toxicological studies for a high percentage of plants [1].

The genus *Solidago* (Asteraceae) includes about 130 plant species worldwide. The different species are wild herbaceous flowering plants used in traditional medicine with anti-inflammatory, diuretic, curative, and antimicrobial effects. These species have bioactive metabolites such as flavonoids, caffeoylquinic acid derivatives, salicylic acid, acid derivatives, saponins, triterpenoids, and diterpenes, among others [2].

One of the species in the *Solidago* genus is *Solidago graminifolia* (syn. *Euthamia graminifolia* (L.) Nutt), an herbaceous perennial plant native to North America, with yellow flowers that often grows from 60 to 150 cm and is easy to grow. Metabolites extracted from the root of *S. graminifolia* have shown pharmacological benefits as bactericidal and fungicidal agents against *Fusarium avenaceum* and *Bipolaris sorokiniana* [3]. Other active components extracted from the root have shown an inhibitory effect on the enzymes α-glucosidase, ß-glucosidase, α-amylase, acetylcholinesterase (*AChE*), and butyrylcholinesterase, showing an antihyperglycemic effect. The aerial parts of the plant have shown activity as an adjuvant in urinary discomfort and a therapeutic effect for diabetes [4]. Additionally, *S. graminifolia* has been reported to have potent cholinesterase inhibitory activity. This activity has been associated with bioactive metabolites obtained from the aerial parts and roots of the plant. Among the most abundant metabolites are flavonoids such as quercetin, chlorogenic acid, rutin, astragalin, terpenes, labdane, diterpenes, and polyacetylenes [4,5,6].

The phytochemical characteristics of this species provide the basis for investigation into inhibitory secondary metabolites of essential proteins for the survival of insect pests, such as acetylcholinesterase (*AChE*) [7]. The insecticidal effect is a minimally explored activity of this plant [8,9].

On the other hand, insect pests in agriculture cause a huge reduction in the quality and yield of crops. In particular, the lepidopteran insect *Spodoptera frugiperda* (*S. frugiperda*) is a pest that has a preference for the consumption of foliage and tender leaves of corn and other plants such as sorghum, grass, sugar cane, rice, beans, cotton, and peanuts [10,11]. *S. frugiperda* is endemic to America; however, it has recently been identified as a migratory pest, invading African and Asian countries and causing losses in agricultural production [12,13]. Therefore, new insecticide molecules are needed to combat this pest.

In this study, the objective was to obtain organic extracts of *S. graminifolia* and evaluate their insecticidal activity against *S. frugiperda* larvae in the laboratory. Additionally, we aimed to identify the secondary metabolites in the extracts by ultra-performance liquid chromatography-mass spectrometry (UPLC-MS) and evaluate the most representative against *S. frugiperda*. Finally, a molecular docking analysis to determine the potential interactions on the active site of *AChE* was performed.

## 2. Results and Discussion

### 2.1. Taxonomic and Molecular Identification

The plant was identified as *S. graminifolia* (syn. *Euthamia graminifolia* (L.) Nutt) with a sequence of 96% homology (744/779) with the MT610936.1 sequence.

### 2.2. Organic Extracts

Table 1 shows the yields obtained from each organic extract. The ethanol (EtOH) extract had a value of 33.49%, two times higher than dichloromethane (DCM) and six times higher than the hexane (Hex) extract. Similarly, high yields using polar solvents such as water, methanol, and EtOH and lower yields with non-polar solvents, such as Hex, have been obtained by other authors [2,14].

### 2.3. Insecticidal Activity of the Extracts

**Figure 1:**
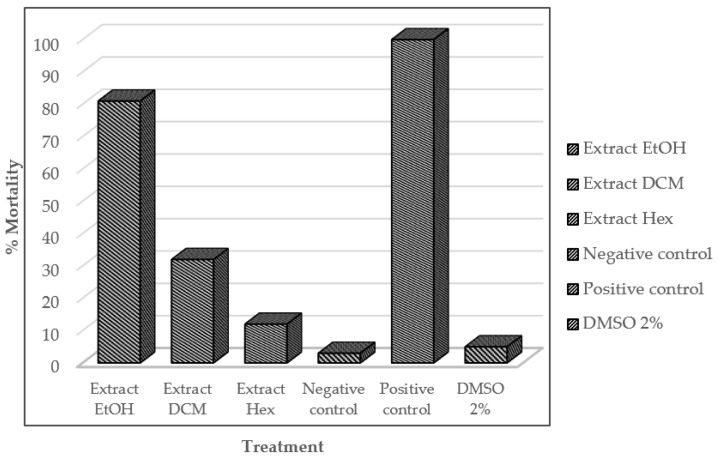
Mortality percentage of organic extracts of *S. graminifolia* against *S. frugiperda* larvae.

The insecticidal activity of the organic extracts against *S. frugiperda* larvae is shown in Figure 1. Higher mortality was found with the EtOH extract (81%); the DCM extract caused 32% mortality, and the Hex extract caused 12%. The commercial insecticide (positive control) had 100% mortality, while the negative control had 3% mortality. Finally, the LC_50_ of the EtOH extract was 0.496 mg/mL.

### 2.4. Phytochemical Analysis

The organic extracts from *S. graminifolia* leaves were analyzed by UPLC to identify their secondary metabolites. Table 2 shows the identified metabolites. The most representative were flavonoids, such as hyperoside, quercetin, isoquercetin, kaempferol and avicularin, and phenolic acids, such as chlorogenic acid, solidagoic acid, gallic acid hexoside, and rosmarinic acid.

Quercetin and solidagoic acid derivatives were two main secondary metabolites identified in the EtOH extract. Both metabolites have been previously described in extracts of *S. virgaurea* as having antibacterial activity [15,17]. Additionally, solidagoic acid has been reported to have insecticidal activity. Therefore, they could be an option for evaluation against *S. frugiperda*.

In the DCM and Hex extracts, a greater presence of quercetin, kaempferol, and chlorogenic acid was identified. Chlorogenic acid is a polyphenol found in plant tissues. Chlorogenic acid extracted from Viburnum opulus and Viburnum lantana has activity against human *AChE* with inhibition percentages of 57.63 ± 1.23% and 87.41 ± 0.99%, respectively. This inhibitory activity of chlorogenic acid has also been described in vivo [18,19]. Quercetin and kaempferol have been described as the main metabolites of *S. virgaurea* L. (European goldenrod, Woundwort) [6]; both metabolites have antioxidant activity [20,21]. A high insecticidal activity has been described for quercetin and other flavonols that act as *AChE* inhibitors, and their possible mechanism of action is associated with the interactions that occur between the flavonol skeleton and the catalytic residues Asp74 or Tyr72 [22,23].

The chlorogenic acid molecule was not detected in the EtOH extract, even though it is described as the most abundant metabolite in plants of the Solidago genus [14,24]. In our case, an overlaping system with many polar metabolites could be the cause of undetected chlorogenic acid. In the species *S. graminifolia*, chlorogenic acid has been identified as the most abundant secondary metabolite in aerial parts extracts with 997.88 ± 7.63 mg/100 g [2].

### 2.5. Insecticidal Evaluation of Secondary Metabolites

Three main secondary metabolites were identified in organic extracts: quercetin, solidagoic acid derivatives, and chlorogenic acid. Quercetin is a flavonoid that has been described as a compound of interest in producing bioinsecticides. Its plant metabolism has been associated with a defense mechanism. The plant alters its palatability and nutritional value, decreases digestibility, or even acts as a toxin [25]. Therefore, quercetin in an EtOH extract could correlate with the highest insecticidal activity [15]. EtOH extract has also shown a high concentration of solidagoic acid derivatives that could cause a biological effect. DCM and Hex extracts have shown lower insecticidal activity with quercetin and chlorogenic acid derivatives, two main secondary metabolites. Chlorogenic acid has been described as an *AChE* inhibitor, an enzyme essential in the hydrolysis of acetylcholine that interrupts transition in the cholinergic synapse of the insect. However, this effect was not reflected in the mortality percentages. It is important to mention that a potential synergism or antagonistic effect could occur with the secondary metabolites in the organic extracts [26].

According to the results of the phytochemical analysis, quercetin and chlorogenic acid with higher relative concentrations in organic extracts were acquired and evaluated to confirm their insecticidal activity. Initially, the LC_50_ of both metabolites was determined. Chlorogenic acid does not show insecticidal activity against *S. frugiperda* larvae, while quercetin has an LC_50_ value of 0.157 mg/mL, a lower concentration than the EtOH extract. Afterward, the percentage of mortality at a concentration of 1 mg/mL was determined in two mixtures to evaluate the synergistic or antagonistic effect of quercetin and chlorogenic acid. In the evaluated mixtures, in a ratio of 1:1, the insecticidal activity decreased, and in a ratio of 1:9, the mixture did not show biological activity (Table 3).

In the quercetin: chlorogenic acid mixture at 1:1 and 1:9, chlorogenic acid caused an antagonistic effect on quercetin and decreased the insecticidal activity against *S. frugiperda* larvae. Quercetin is a promising molecule as an insecticidal agent, with interesting biological and ecofriendly aspects. Quercetin has harmful effects on the development and body weight of larvae belonging to noctuid insects, but selectively, since it has shown activity against Hemiptera, Diptera, and Lepidoptera, but is less harmful to Coleoptera. This finding suggests that the quercetin molecule allows herbivore control without disrupting the role of natural enemies and pollinators [27]. Additionally, quercetin may generate a synergistic relationship with a possible inhibitory activity of oxidases associated with the detoxification of insecticides used by insect larvae through glutathione-S-transferase [28].

### 2.6. Molecular Docking Analysis of Quercetin on S. frugiperda Acetylcholinesterase

**Figure 2:**
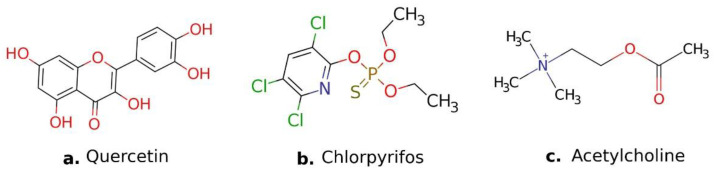
(**a**) Structure of quercetin, main metabolites of *S. graminifolia*; (**b**) chlorpyrifos, an organophosphate insecticide; (**c**) acetylcholine, the natural substrate for AchE.

To determine the possible mechanism of action of quercetin, a molecular docking analysis on the active site of *S. frugiperda AChE* was performed. The natural substrate acetylcholine was used as a reference compound, which participates in the inhibition of *AChE* and is considered one of the most effective molecules for the restoration of the cholinergic system [22]. Another reference molecule used was the organophosphate insecticide chlorpyrifos, which acts as an *AChE* blocker in nerve endings, generating an accumulation of acetylcholine and consequently, an alteration in the functioning of the nerve impulse. Both were used with the aim of comparing the interactions involved on the active site of *S. frugiperda AChE* [29]. The chemical structures are shown in Figure 2. 

**Figure 3:**
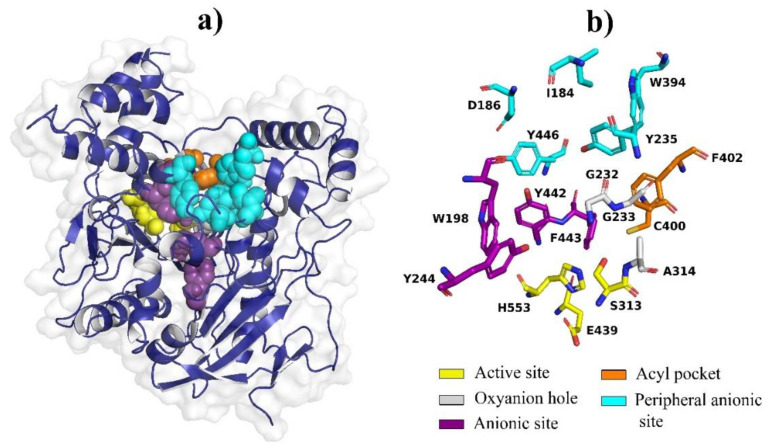
Homology modeling of the *S. frugiperda AChE* protein. (**a**) Prediction of the 3D structure of *AChE* and (**b**) identification of amino acid residues on the active site.

The 3D structure of *S. frugiperda AChE* is not available in the Protein Data Bank. For this reason, modeling the protein by homology was performed. This model is based on the 3D prediction of structures from evolutionary-related proteins, selecting the template 6ARX (2.30 Å) with an identity of 71.27% with respect to the amino acid sequence of S. exigua AchE. The model presents standard deviations with a GMQE of 0.62 and a QMEAN of −0.48. Additionally, the *AChE* model was evaluated with PROCHECK [30]. The results show that 89.6% of the residues are in favored regions, 9.5% in additionally allowed regions, 0.7% in generously allowed regions, and 0.2% in disallowed regions, indicating that the arrangement of the dihedral angles is correct. The result of modeling based on the 6ARX template is presented in Figure 3.

**Figure 4:**
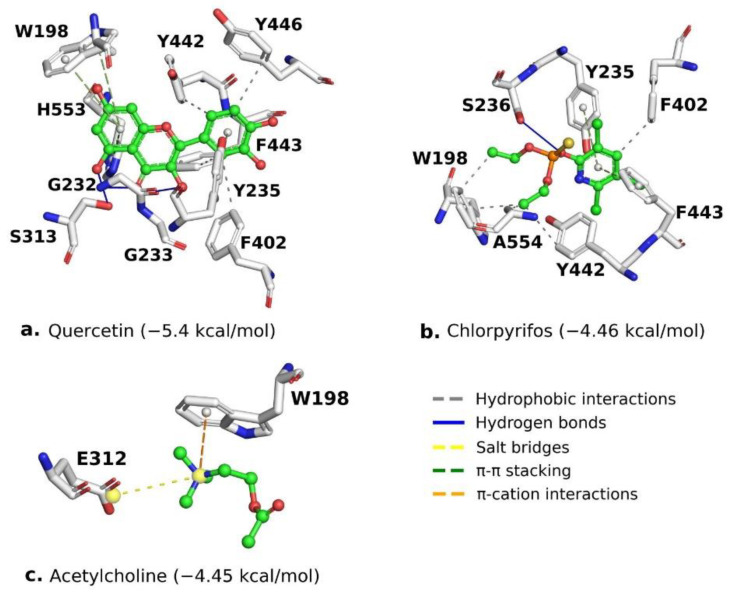
Interaction profile of the compounds in the modeled *AChE* protein. (**a**) Quercetin, (**b**) chlorpyrifos, and (**c**) acetylcholine.

Chlorpyrifos (−4.46 kcal/mol) had four hydrophobic interactions (W198, F402, Y442, A554) that include amino acids from the anionic site and the acyl cavity, a hydrogen bond, and two π–π stacking interactions (Y235 and F443) from the peripheral anionic sites. Acetylcholine (−4.45 kcal/mol) showed only two types of interactions: a salt bridge and a π–cation interaction. Both controls presented similar binding energies and were not higher than the test compound. Quercetin showed a value of −5.4 kcal/mol. Quercetin had three types of interactions: hydrophobic (F402, Y442, F443, Y446), which are residues of the acyl cavity, the anionic site, and the peripheral anionic site; hydrogen bonds with the oxyanion cavity (G232, G233) and the active site (S313); and π–π stacking with the amino acids W198, Y235, and H553. The interactions described above are shown in Figure 4.

**Figure 5:**
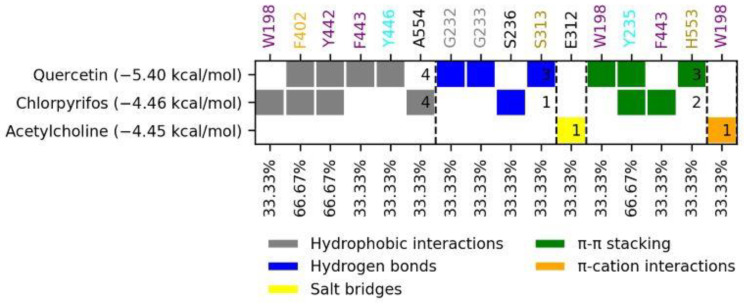
Fingerprints of quercetin interaction and control compounds on the active site of *S. frugiperda AChE*.

The better insecticidal activity of quercetin can be regulated by interactions on the active site of *AChE* (Figure 5). These interactions have a unique nature that can enhance its protein binding capacity [31]. It has been described in flavonoid derivatives such as quercetagetin, whose hydrogen bond interactions can reduce the force of interaction but positively enhance this molecule’s antioxidant activity [32].

## 3. Materials and Methods

### 3.1. Collection of Plant Material

Eight whole plants (approximately 60 cm high) were collected from November 2020 to July 2021 in the city of Villa de Cos, state of Zacatecas, Mexico, coordinates 23°16′32.0″ N–102°14′57.4″ W. The specimens were transferred to the Laboratory of Chemistry-Biochemistry at the Unidad Académica Multidisciplinaria Mante of the Universidad Autónoma de Tamulipas for the preparation of organic extracts and molecular identification.

One specimen was placed in a botanical press and sent to the Instituto de Ecología Aplicada of the Universidad Autónoma de Tamaulipas for genus and species identification by consulting specialized botanical literature and specialists from the Asteraceae family. The Voucher specimen was deposited in the herbarium of Francisco González Medrano at the same institution, with the code UAT-22866.

### 3.2. Molecular Identification

The fresh leaves of the collected specimens were separated, and 200–500 mg were kept at −70 °C until use. Genomic DNA was extracted using the Wizard Genomic DNA Purification Kit (PROMEGA, Madison, WI, USA) following the protocol for Isolating Genomic DNA from Plant Tissue. The internal transcribed spacer (ITS) region of 18S–26S nuclear ribosomal DNA (rDNA) was amplified by PCR, using the primers reported by [33] for the Asteraceae family. The primers were designed on a conserved sequence outside the ITS region; the primer ITS-20F 5′-TCGCGTTGACTACGTCCCTGCC-3′ was located 200 bp from the 5′ of ITS-1 region, while ITS-262R 5′-ATTCCCAAACAACCCGACTCG-3′ was 250 bp from the 3′ region of ITS-2.

The PCR reaction was carried out using 50 ng of DNA, 1.5 mM of MgCl_2_, 0.2 mM of dNTP, 0.5 mM of each primer, and 0.05 U of Taq polymerase, using the following temperatures: a cycle of 94 °C for 5 min, 35 cycles of 94 °C for 30 s, 57 °C for 30 s, 72 °C for 1 min, and a final cycle of 72 °C for 7 min. The PCR product was sent for sequencing to Eurofins (USA), using 15 µL of the PCR product at a concentration of 5 ng/µL and 2 µL of the primer ITS-20F at a concentration of 10 pmol/µL. Using BLAST software (https://blast.ncbi.nlm.nih.gov, accessed on 10 September 2021), the sequence obtained was aligned with the nr database of the NCBI to identify the plant genus and species.

### 3.3. Preparation of Organic Extracts

The leaves were separated and placed to dry in a Felisa oven at 60 °C until they reached a constant weight; they were then pulverized manually. Solvents, ethanol (EtOH), dichloromethane (DCM), and hexane (Hex) were used in a gradient of polarity to obtain plant extracts; 100 g of leaves in 500 mL of the corresponding solvent were subjected to constant agitation for a week in dark conditions. Afterward, the samples were vacuum-filtered, and the solvent was eliminated in a rotary evaporator (Heidolph) to obtain the crude extract.

### 3.4. Ultra-Performance Liquid Chromatography (UPLC)

The organic extract samples were analyzed using the following procedure: 1 mg of extract was previously weighed and dissolved in 1 mL of HPLC-grade solvent used in the initial extraction (ethanol, dichloromethane, and hexane) and filtered through a 0.45 µm syringe filter for analysis. UPLC-MS/MS analyses were carried out using an ACQUITY UPLC system coupled to a Waters QDA^®^ mass detector (Milford, MA, USA). The ion transitions monitored were 559–440 Da. The cone potential (15 V) was the optimum value for positive-ion mode, and the capillary potential was 1.5 kV. ACQUITY UPLC CORTECS^®^ C18 1.6 µm 3.0 column × 100 mm in positive-ion mode with a column temperature of 40 °C, and an autosampler temperature of 15 °C. Elution was achieved with 0.1% formic acid in water (Phase A), acetonitrile (Phase B), and 5 mM ammonium acetate (Phase C). The flow rate was 0.3 mL/min, and the injection volume was 5 μL. The composition of the solvents over time were initial A: 5%; B: 85%; C: 10%, at 3.0 min increase A: 15%; B: 75%; C: 10%, changing at 10.0 min to A: 5%; B: 85%; C:10%. The running time was 15.0 min.

### 3.5. Insecticidal Activity

The third and fifth instar of *S. frugiperda* larvae were collected from maize crops at the Experimental Agricultural field of the Unidad Académica Multidisciplinaria Mante in Cd. Mante, Tamaulipas, 22°43′03.5″ N 98°57′50.4″ W. The larvae were individually placed in plastic cups number zero with a lid to avoid losses due to cannibalism; later, they were transferred to the insect breeding room of the botanical laboratory. The larvae were individually placed in containers with an artificial diet (100 mL of water, soy flour 7.1 g, wheat germ 3.1 g, yeast 1.0 g, Wesson salt 1.0 g, agar agar 1.0 g, sorbic acid 0.2 g, methyl paraben 0.25 g, ascorbic acid 0.4 g, choline chloride 0.2 g, formaldehyde 40% 0.25 mL, and Vanderzant vitamins 0.2 g) under 12:12 light/dark photoperiod conditions and at a temperature of 30 °C with 60% relative humidity (RH). The establishment of the breeding stock was carried out until the F_2_ generation to rule out deaths due to parasitism or another disease. Their biological cycle was followed until egg hatching was achieved. For the bioassays, 2-day-old neonate larvae were used for all treatments [7].

The mortality percentage (%) of the organic extracts (EtOH, DCM, and Hex) was determined at a concentration of 1 mg/mL by an ingestion bioassay. Subsequently, the half-maximal lethal concentration (LC_50_) of the EtOH extract was determined. The evaluation was carried out through ingestion bioassays using seven treatments: four concentrations of EtOH extract at concentrations of 1.0, 0.5, 0.25, and 0.1 mg/mL incorporated into the diet; three control treatments—two negatives, the first using only diet and the second using a diet with 2.0% DMSO, as it was the concentration used to dissolve the extracts; the third treatment was the commercial insecticide (Chlorpyrifos S 480) as a positive control at the dose recommended by the manufacturer. Each extract was homogenized with a stirring plate in 25 mL of artificial diet. Once the artificial diet solidified, diet pieces 0.5 cm long and 0.5 cm wide were cut. The cut pieces were placed individually in number zero plastic cups, where each two-day aged *S. frugiperda* larvae was placed. Twenty-five larvae were used for each treatment. The test was carried out in triplicate, obtaining a total population of 75 larvae per treatment, maintained in 12:12 light/dark photoperiods at a temperature of 30 °C with 60% RH for 120 h. After the 120 h period, mortality was recorded for each treatment, considering a larva that did not move or react when touched with a camel hairbrush dead. The LC_50_ was determined with a Probit analysis in the SPSS statistical program with a significance level of 0.05.

### 3.6. Molecular Docking Analysis

Homology modeling of the *AChE* type I protein was performed using the amino acid sequence of *Spodoptera exigua* (GenBank AZB49078.1). With the MUSCLE tool [34], a 99.38% identity was verified with the partial sequence of the *AChE* protein from *S. frugiperda* (GenBank AGK44160.1). The amino acid sequence of *AChE* was entered in FASTA format into the Swiss Model platform (https://swissmodel.expasy.org, accessed on 20 January 2022) to predict the 3D structure of the protein. The template selection for modeling was made according to the percent identity, protein resolution, global model quality estimation (GMQE), qualitative model energy analysis (QMEAN), range, and protein coverage [35]. The modeled *AChE* was evaluated using PROCHECK [30] to determine the geometric quality of the φ and ψ angles of the amino acids in the protein.

The modeled *AChE* was prepared in the UCSF Chimera v1.15 program [36], removing the additional A chain molecules. Polar hydrogens were added to the Dock Prep module, and the side chains were repaired. Finally, the protein was converted to PDBQT format with AutoDock Tools 1.5.6 [37]. Subsequently, the residues on the active site of the modeled protein were determined by 3D alignment (Protein structure comparison service PDBeFold at EBI) with the *AChE* of *Torpedo californica* (PDB: 6G1U). The selected ligands for predicting the inhibitory activity against *AChE* were quercetin and chlorogenic acid. Acetylcholine and chlorpyrifos were considered controls.

The compounds were drawn using the MarvinSketch v21.4 program (http://www.chemaxon.com, accessed on 20 January 2022); later, the molecules were energetically minimized, and the polar hydrogens were added with OpenBabel 3.1.1. Finally, the ligands were converted to PDBQT with the Gasteiger charge with prepare_ligand.py from AutoDock Tools. Molecular docking was carried out using AutoDock Vina 1.1.2. The center of the box was determined on the active site of *AChE* modeled at X = −59.961 Å, Y = 58.987 Å, and Z = 22.919 Å, and the box dimension was 20 Å in XYZ. The interaction analysis of the couplings was performed using Protein-Ligand Interaction Profiler (PLIP) software.

### 3.7. Insecticidal Activity of Chlorogenic Acid and Quercetin

Chlorogenic acid and quercetin (Sigma-Aldrich^®^, St. Louis, MO, USA, with a purity ≥98%) were evaluated by ingestion bioassays. The compounds were added to 25 mL of diet adjusted to concentrations of 1.0, 0.5, 0.25 and 0.1 mg/mL. Additionally, two treatments were evaluated at 1.0 mg/mL to determine the synergism between chlorogenic acid and quercetin. The treatments had a ratio of 1:1 and 9:1 [2]. The evaluation was drawn from the ingestion bioassay following the methodology described above. Finally, the LC_50_ was determined with a Probit analysis in the SPSS statistical program with a significance level of 0.05.

## 4. Conclusions

In this study, an in vitro insecticidal assay of three organic extracts of *S. graminifolia* showed that these extracts cause mortality in *S. frugiperda*. The EtOH extract had the highest biological effect with a value of 81% and an LC_50_ of 0.496 mg/mL. The phytochemical analysis showed that the organic extracts have two main secondary metabolites that are potentially responsible for the biological effects: quercetin and chlorogenic acid. Quercetin had an insecticidal activity with a LC_50_ value of 0.157 mg/mL, and chlorogenic acid did not show insecticidal activity. However, both secondary metabolites had an antagonistic effect: in a 1:1 ratio, the LC_50_ value decreased to 0.729 mg/mL, and in a 9:1 ratio, the insecticidal effect was annulled. Finally, the molecular docking analysis suggests that the mechanism of action of quercetin is as an inhibitor of *S. frugiperda AChE* with better values of binding energy (−5.4 kcal/mol) than acetylcholine and chlorpyrifos (−4.45 and −4.46 kcal/mol, respectively). These results suggest that quercetin could be a new option to develop more botanical insecticidal agents.

## Figures and Tables

**Table 1:** Percentage yield from organic extracts of *S. graminifolia* leaves (100 g).

Solvent	Yield (%)	mg per g
Ethanol	33.39	333
Dichloromethane	18.34	183
Hexane	5.03	50

**Table 2:** Secondary metabolites from organic extracts of *S. graminifolia* identified by UPLC-MS.

Extract	Compound	Molecular Formula	Theoretical *m*/*z*	Experimental *m*/*z*	Reference
EtOH	Solidagoic acid G	C_21_H_30_O_5_	361.19	361.13	[15]
	Unknown	--	--	542.23	--
	Quercetin	C_15_H_10_O_7_	302.23	303.28	[2] [6]
	Solidagoic acid C	C_20_H_28_O_4_	331.0	331.10	[15]
	Unknown	--	--	104.02	--
DCM	Unknown	--	--	377.11	--
	Quercetin	C_15_H_10_O_7_	302.23	303.28	[2] [6]
	Unknown	--	--	379.25	--
	Solidagoic acid B	C_25_H_34_O_5_	414.5	415.18	[15]
	Rosmarinic acid	C_18_H_16_O_8_	360.3	360.41	[2]
	Chlorogenic acid	C_16_H_18_O_9_	355.00	356.45	[2]
Hex	Solidagoic acid B	C_25_H_34_O_5_	414.5	415.24	[15] [16]
	Unknown	--	--	102.04	--
	Hyperoside	C_21_H_20_O_12_	464.4	465.27	[2] [6]
	Quercetin	C_15_H_10_O_7_	302.23	303.21	[2] [6]
	Unknown	--	--	407.28	--
	Unknown	--	--	389.25	--
	Chlorogenic acid	C_16_H_18_O_9_	355.00	356.23	[2]

**Table 3:** Insecticidal activity of quercetin and chlorogenic acid and their mixture in two ratios.

Compounds	CL_50_ (mg/mL)	Mixture of Compounds	CL_50_ (mg/mL)
Quercetin	0.157	Quercetin: Chlorogenic acid 1:1	0.729
Chlorogenic acid	No insecticidal activity	Quercetin: Chlorogenic acid 1:9	No insecticidal activity

## Data Availability

Available on reasonable request to the corresponding author.
